# A case of rare gastric metastasis of invasive lobular carcinoma of the breast

**DOI:** 10.1093/jscr/rjad142

**Published:** 2023-03-14

**Authors:** Yicong Liang, Steven R Paredes, Josh Karpes, Bruce T C Chau, Frank Wang

**Affiliations:** Department of General Surgery, Campbelltown Hospital, Sydney, NSW 2560, Australia; School of Medicine, University of New South Wales, Sydney, NSW 2033, Australia; Department of General Surgery, Campbelltown Hospital, Sydney, NSW 2560, Australia; Department of General Surgery, Campbelltown Hospital, Sydney, NSW 2560, Australia; Department of Anatomical Pathology, Liverpool Hospital, Sydney, NSW 2170, Australia; Department of General Surgery, Campbelltown Hospital, Sydney, NSW 2560, Australia; School of Medicine, Western Sydney University, Sydney, NSW 2751, Australia

**Keywords:** breast cancer, invasive lobular carcinoma, gastric metastasis

## Abstract

Breast cancer is the most common type of cancer diagnosed among women worldwide. It significantly contributes to cancer-related mortality in females. Early-stage breast cancers have a high cure rate. However, distant metastasis of breast cancer due to haematological and lymphatic spread often leads to a poor prognosis. Gastric and duodenal metastasises are rarely observed in invasive lobular carcinoma of the breast. Early diagnosis is challenging due to the non-specific symptoms, the limited specificity of radiological investigations and the difficulty in obtaining adequate tissue biopsy. Herein, we report the clinical, radiological, and macroscopic features of a 72-year-old female with rare gastric metastasis of breast cancer and likely concurrent duodenal metastasis.

## INTRODUCTION

Breast cancer is the second most common cancer worldwide and the most common cause of cancer-related mortality among females [1]. Metastasis of breast cancer to the gastrointestinal (GI) tract is rare. The colon and rectum are the most common sites of gastrointestinal tract metastasis, followed by stomach and small intestine [2, 3]. Herein, we report a case of a 72-year-old female with rare gastric metastasis of breast cancer, followed by a discussion of difficulties and challenges in the diagnosis of this condition.

## CASE REPORT

A 72-year-old female was diagnosed with 3 weeks of vomiting associated with dull right-sided abdominal pain and 5 kg weight loss in four weeks. The patient had a history of stage IIIc (pT2N3) multifocal left pleomorphic lobular carcinoma diagnosed two years prior to admission with axilla lymph node metastasis. She underwent a left mastectomy and axillary lymph node dissection, followed by adjuvant radiotherapy to the left chest wall, axilla, the supraclavicular fossa and the internal mammary chain (42.4Gy/16 fractions). The patient declined chemotherapy due to her frailty (ECOG score = 2–3) and high-risk comorbidities.

**Figure 1:**
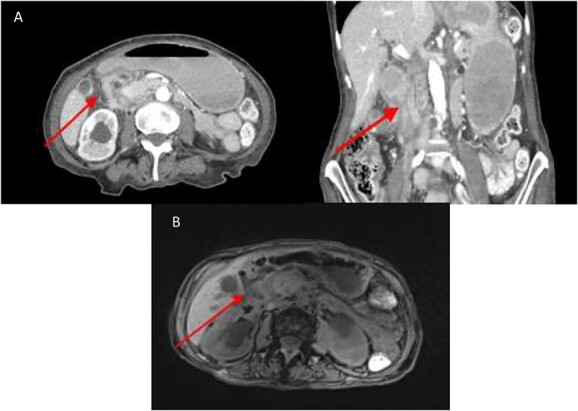
(**A**) CT abdomen showing marked luminal narrowing (red arrow) at the second part of the duodenum (axial and coronal section). (**B**). T1 MRI Showing retroperitoneal soft tissue (red arrow) paralleling the second part of duodenum resulting in extrinsic compression.

An abdominal computed tomography (CT) performed at this admission demonstrated soft tissue thickening around the second part of duodenum (D2), suggestive of a possible malignant stricture (Fig. 1A). Magnetic resonance imaging (MRI) showed an area of retroperitoneal soft tissue paralleling D2 resulting in extrinsic compression (Fig. 1B).

**Figure 2:**
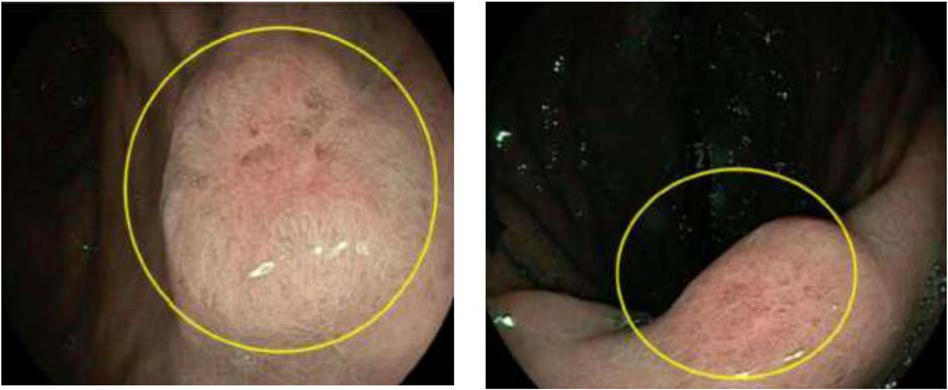
Nodules with unusual surface vascular pattern in found in gastric antrum (left) and incisura (right) at the time of endoscopy—metastatic tissue of breast origin confirmed by biopsy.

**Figure 3:**
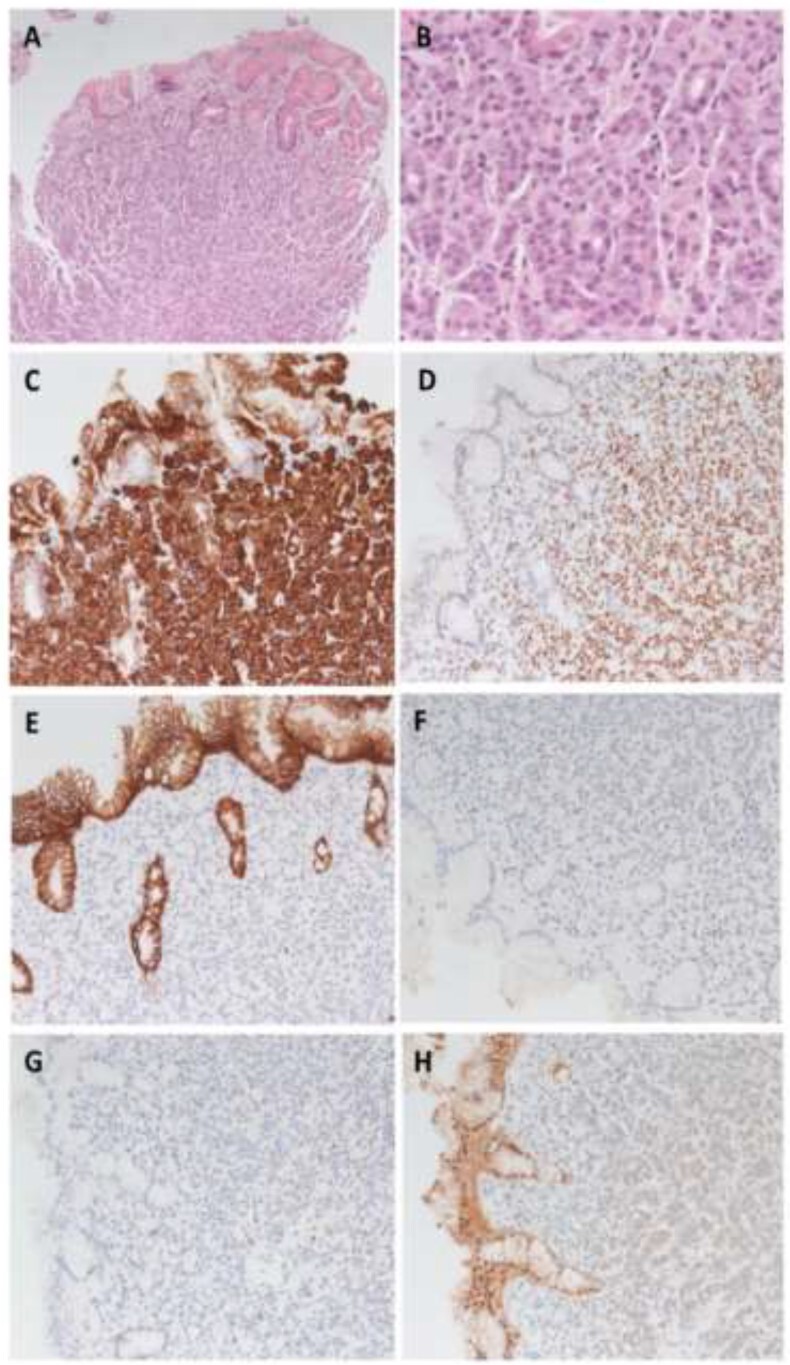
Metastasis histology and immunohistochemistry. (**A**) Photomicrograph of lobular carcinoma metastasis with infiltration into gastric mucosa (HE 100×). **B**: High power photomicrograph of metastatic tumour cells (HE 400×). (**C**)–(**D**): Tumour cells are positive for CK7 (C) and GATA3 (D), consistent with breast primary origin (200×). (**E**) Loss of e-Cadherin staining within tumour cells in contrast to gastric epithelium supporting invasive lobular carcinoma (200×). (**F**)–(**H**) Metastatic tumour cells are negative for oestrogen receptor staining (F), progesterone receptor staining (G) and equivocal (2+) for HER2 staining (H) (200×).

A fluorodeoxyglucose positron emission tomography (FDG-PET) scan did not demonstrate definite evidence of local recurrence. There was no increased uptake in the region surrounding D2, although FDG-avidity was observed in a right crural lymph node. Gastroscopy identified a nodule in the gastric antrum with an unusual vascular surface pattern (Fig. 2). The first two parts of the duodenum showed congested and oedematous mucosa. The endoscope could not traverse the stenosis in D2 thus precluding biopsy of the D2 segment. The histopathologic analysis of the gastric biopsy confirmed the presence of a metastatic carcinoma within the lamina propria of the stomach, with morphology and immunophenotype strongly favouring primary site of origin from the breast. The metastatic carcinoma stained oestrogen receptor negative, progesterone receptor negative and HER 2 equivocal (+2 membranous staining intensity) (Fig 3). HER-2 *in situ* hybridisation was not performed to confirm HER2 copy number secondary to diagnostic rather than treatment intent of the biopsy. The duodenal biopsy contained no abnormalities.

It was hypothesized that the duodenal obstruction was caused by metastases from the recurrence of invasive lobular carcinoma of the breast. However, a definitive histopathological diagnosis was not possible as both radiological and surgical approaches to biopsying the paraduodenal lesion were deemed too high-risk. Treatment options were subsequently discussed at a multidisciplinary team meeting and systemic, endoscopic, laparoscopic and open surgical options were considered. The patient was too frail for palliative chemotherapy. The benefits of endoscopic stenting were outweighed by the risks of stent migration, erosion and obstruction. The patient and her family declined any surgical intervention. Palliative radiotherapy was the most appropriate treatment given the circumstance.

## DISCUSSION

Diagnosis of gastrointestinal metastasis of breast cancer can be challenging. Patients usually present with non-specific symptoms including epigastric pain, nausea, vomiting, dyspepsia and dysphagia [4]. The similarity of the obstructive symptoms to other primary gastrointestinal patholog often lead to a delay in diagnosis and possible confusion with primary gastric malignancy. Furthermore, gastric metastases of breast cancer usually present as part of widespread disseminated disease. Although gastric metastases of invasive lobular carcinoma of the breast has been previously documented, it is rare for breast cancer to manifest in the stomach as the first site of distant metastasis [5, 6]. In this case, the time between the diagnosis of primary breast cancer and the distant metastasis was approximately two years. There was no evidence of widespread disease and the metastasis was localized to one segment of the GI tract. It should be noted that the patient only received local treatment for the primary tumour, which was oestrogen receptor negative with lymph node involvement. These factors have been demonstrated to confer an earlier and greater risk of distant metastatic recurrence that peaks at approximately 2 years compared with breast cancers without these histological features (peaking between 6 and 10 years) [7].

The radiological distinction between primary gastric cancer and breast cancer with gastric metastasis is difficult. Abdominal CT and barium study findings are usually non-specific. PET CT is reported to have a low sensitivity in diagnosing the origin of metastasis. However, it could be useful in assessing treatment response [4]. Endoscopic visual assessment alone has limited utility in making the diagnosis of gastric metastasis of breast cancer as it is often difficult to distinguish these lesions from the primary gastric cancer macroscopically [8].

Biopsy and subsequent immunohistochemical analysis remain the most accurate method in differentiating between metastatic breast cancer and primary gastric cancer. However, the initial gastric biopsies could be reported as normal in 46–50% of cases as invasion of malignant cells are limited to the submucosa and the seromuscular layers and can be missed with superficial sampling (biopsy sampling encompassing the lamina propria only). Therefore, deep and extensive biopsies are recommended [4, 9]. The limitation of endoscopic biopsy was observed in our case as the initial superficial duodenal biopsy was negative despite the high likelihood of metastatic breast cancer compressing the duodenum on the basis of the clinical presentation and the radiological findings. Furthermore, it has also been reported that, amongst the different breast cancer subtypes, invasive lobular carcinoma more commonly metastasises to the GI tract, although the specific underlying pathogenesis remains uncertain [9].

In conclusion, we reported a rare case of gastric metastasis of invasive lobular carcinoma of the breast. The rarity of breast cancer with gastric metastasis, the non-specific symptoms associated with the gastric metastasis, the low specificity of the radiological imaging and the difficulty in obtaining adequate tissue biopsies are challenges hinder early diagnosis. As a result, the possibility of gastric metastasis should not be overlooked in patients with a history of ILC of the breast presenting with a suspicious gastric or duodenal lesion.
